# Not Just a Fluke: A One Health review on *Schistosoma japonicum* and *Fasciola* spp*.* in Malaysia

**DOI:** 10.1016/j.onehlt.2026.101612

**Published:** 2026-10-13

**Authors:** Jessica Wyn Brocklebank, Luke Davies, Benoît Goossens, Laura Braun, Pablo Orozco-terWengel

**Affiliations:** a School of Biosciences, Cardiff University, Cardiff, United Kingdom; b Danau Girang Field Centre, c/o Sabah Wildlife Department, Kota Kinabalu, Malaysia; c London School of Hygiene and Tropical Medicine, London, United Kingdom

**Keywords:** Schistosomiasis, Zoonotic parasites, Integrated control strategies, Fascioliasis, Livestock, Transmission ecology, Helminth

## Abstract

Zoonotic trematodes *Schistosoma japonicum*, *Fasciola hepatica* and *Fasciola gigantica* are neglected tropical diseases imposing significant health and livestock burdens in Asia. Control remains uneven due to complex host–parasite–environment interactions and fragmented surveillance. Both species utilise aquatic snail intermediates and hepatobiliary tropism, presenting common challenges for diagnosis, treatment, and control. Agricultural intensification and landscape change reshape human–livestock–environment interfaces, facilitating transmission. Despite progress, diagnostics remain constrained by low sensitivity, variable performance, and limited scalability. This review synthesises advances in parasite biology, transmission ecology, and socioecological drivers, with Malaysia as an illustrative case, and identifies One Health opportunities. We evaluate emerging integrated strategies, persistent barriers, and outline priorities for harmonised diagnostics, strengthened multisectoral surveillance, and community-centred interventions.

## Highlights

• Synthesises zoonotic flukes *S. japonicum* and *Fasciola* spp. via One Health.
• Reveals shared transmission drivers across livestock, snails and landscapes.
• Exposes critical surveillance gaps masking Malaysia’s true fluke burden.
• Benchmarks diagnostic tools across human and veterinary health sectors.
• Outlines feasible, integrated One Health control pathways for Malaysia.

## 1 Flukes: Ecology, Lifecycle & Clinical Features

Zoonotic diseases are defined as diseases transmitted between non-human animals and humans. Being highly prevalent worldwide, 58% of all known human pathogens are of zoonotic origin [1], [2], [3]. Moreover, of all emerging infectious diseases, 60% are classified as zoonotic, with most linked to mammal hosts or intermediate species [4], [5]. Worldwide, zoonotic diseases cause approximately one billion cases of illness in humans annually, leading to millions of fatalities [6]. Zoonotic disease transmission and distribution has surged in recent centuries, driven by global connectivity and human pressures on ecosystems [7].

*Schistosoma japonicum*, alongside *Fasciola hepatica and Fasciola gigantica* (referred to hereafter as *Fasciola* spp.,) are major zoonotic trematodes with similar hepatobiliary tropism (targeting of the liver, gallbladder and bile ducts), host–parasite dynamics, and symptomology (Table 1) [8], [9]. This poses shared challenges for diagnosis, control, and treatment. These parasites are a neglected tropical disease within the broader framework of zoonotic diseases. Moreover, their transmission cycles (Fig. 1) involve animal hosts (livestock) that are in close association with human populations, complicating parasite eradication efforts. Adding to this are the changes in distributions of intermediate hosts (aquatic snails), due to socioecological conditions of the twenty-first century [7], [10], [11]. This has generated ample opportunities for zoonotic pathogens spillover, thereby enabling them to become a global threat [7], [10], [11]. Many of these socioecological changes such as land-use conversion, agricultural intensification, and water management directly shape critical stages of *S. japonicum* and *Fasciola* spp*.,* lifecycles. Deforestation and irrigation create stagnant water bodies that support snail hosts e.g. *Robertsiella, Oncomelania or Radix* spp*.* which are all known to host *S. japonicum* across Asia, *or Lymnaeidae* spp., which are hosts of *Fasciola* spp*.*, enabling miracidia from eggs deposited in faeces from livestock or humans to penetrate and develop within snails [12], [13], [14]. Agricultural practices like rice cultivation also sustain snail populations, promoting sporocyst development and cercarial shedding into water sources [15]. Human and livestock contact with these waters during farming or bathing practices facilitates skin penetration by *S. japonicum* cercariae, while aquatic vegetation in irrigated fields provides substrates for metacercarial encystment of *Fasciola* spp*.*, increasing infection risk through ingestion [16]. In Southeast Asia, these ecological drivers intensify human–animal–water contact, reinforcing parasite interactions and highlighting an urgent need for integrated surveillance and control strategies [17]. Although *Opisthorchis viverrini* and *Clonorchis sinensis* exhibit similar lifecycles involving aquatic snail intermediate hosts and geographic distributions, this review focuses on trematode species that pose significant risks at the interface of terrestrial livestock production and human health.

**Table 1:** Comparative overview of *Schistosoma japonicum* and *Fasciola* spp*.,* in a One Health context.

Species	Morphology	Intermediate host(s)	Preferred Anatomical Site	Definitive host(s)	Clinical features (humans & animals)	Human–water–animal interactions	Zoonotic & public health significance
*Schistosoma japonicum*	Slender, cylindrical dioecious trematode. Males ∼12 × 0.5 mm; females ∼20 × 0.4 mm [18], [19]. Females produce ∼3,500 eggs per day [20], [21].	*Oncomelania, Robertsiella* and *Radix* spp. snails [13], [14], [22], [23].	Mesenteric venous plexus [24], [25]	Humans and >40 mammalian reservoir species (e.g. bovines, buffalo, rodents, dogs) across East and Southeast Asia [18], [19].	**Humans:** Often asymptomatic (up to 42%) [26]. Cercarial dermatitis within 24 h in a minority (7–36%) [26]. Acute phase (8–12 weeks): Katayama fever (fever, urticaria, cough, eosinophilia) [22], [27]. Chronic disease: RUQ pain, hepatosplenic schistosomiasis, portal hypertension, ascites, splenomegaly, oesophageal varices, GI bleeding, encephalopathy [28]. **Animals (ruminants):** Initially asymptomatic, chronic infections can cause reduced productivity, increased susceptibility to secondary infections, anaemia, hypoalbuminemia, hyperglobulinemia, and marked eosinophilia [29], [30].	Close coupling of livestock grazing, freshwater habitats, and human water contact. Water buffalo and cattle contaminate water bodies with eggs via faeces during wallowing, grazing, or draught work, sustaining snail infection [30], [31], [32]. Humans acquire infection through agricultural, pastoral, and domestic water contact (e.g. rice farming, irrigation, livestock watering) [17], [33], [34].Presence of large mammalian reservoirs makes shared water use a critical driver of transmission [35], [36], [37].	Infects ∼1-2 million people across Asia annually [38]. Extensive animal reservoirs sustain transmission, complicating control [30].
*Fasciola hepatica & Fasciola gigantica*	Leaf-shaped, spiny, monoecious (hermaphroditic) trematode [39]. Produce upwards of 20,000 eggs per day [40] ***F. hepatica***: ∼30 × 15 mm [39], [41]. ***F. gigantica***: ∼25–75 mm×12 mm [9], [39]	*Lymnaeidae snails* (e.g. *Galba*, *Lymnaea*) [42], [43].	Intra and extrahepatic bile ducts [39], [41]	Humans and mammalian hosts; Ruminants (sheep, cattle, goats, buffalo) [39].	**Humans:** Up to 50% asymptomatic [39]. Acute phase (8–12 weeks): RUQ pain, fever, urticaria, asthenia, weight loss due to hepatic migration. Chronic phase: biliary obstruction, hepatomegaly, liver lesions, fibrosis/cirrhosis. **Animals (ruminants):** Acute phase characterised by sudden onset of hepatitis due to migrating juvenile flukes, characterised by lethargy, anorexia, abdominal pain, anaemia, haemorrhage, and, in heavy infections, sudden death [30], [39]. Chronic fascioliasis: Reduced weight gain and carcass quality, anaemia with hypoproteinaemia (“bottle jaw”), poor body condition, reduced fertility, and increased susceptibility to secondary infections [30], [39].	Transmission driven by shared grazing landscapes and freshwater vegetation [44], [45]. Livestock shed eggs into wet pastures and waterways; metacercariae encyst on aquatic plants consumed by ruminants or humans [39], [41], [46]. Water buffalo and cattle amplify environmental contamination through constant pasture–water interface use [15], [30]. Humans are exposed via handling livestock, irrigation, and consumption of raw aquatic plants, linking agricultural water use with zoonotic risk [44], [46], [47].	∼2.4 million human cases annually; ∼180 million people and >300 million livestock at risk in >80 countries [39], [48]. Major economic losses in agriculture [30].

Abbreviations as follows: GI, gastrointestinal; RUQ, right upper quadrant.

**Fig. 1:**
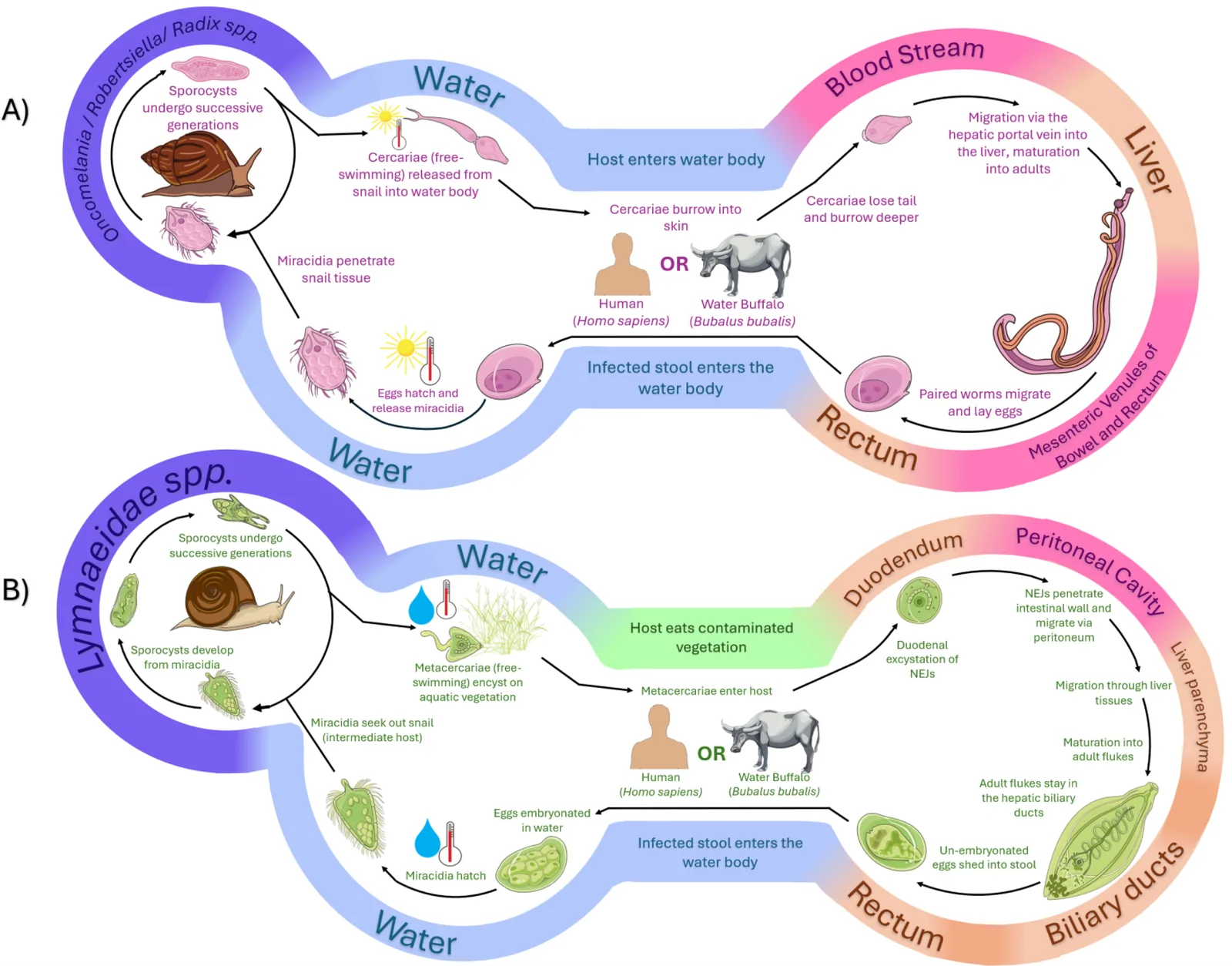
The ‘One Health’ Lifecycle of *Schistosoma japonicum, and Fasciola* spp*.* *A) Schistosoma japonicum* lifecycle *B) Fasciola* spp. lifecycle Colourings are as follows: Blue = aquatic / water body, dark purple = intermediate host, green = in or on vegetation, pink = blood/ blood vessels, orange = organs. Icons represent the following in stages of development: Thermometer = heat dependent stage, Sun = light dependent stage, Water Drop = humidity dependent stage. Water buffalo are used here as an example of one of the many livestock species that *S. japonicum,* and *Fasciola* spp*.* may infect. Abbreviations as follows: NEJ = newly excysted juveniles. Figure created using elements from Servier Medical Art [49] and Integration and Application Network [50].

## 2 One Health Dynamics of Fluke Epidemiology: Global Trends & Risk Factors

Current classifications list *S. japonicum* as non-endemic in Malaysia, with the locally occurring *Schistosoma malayensis* considered of greater public health relevance [23]. In contrast, both *F. hepatica* and *F. gigantica* are recognised as endemic, particularly within livestock [15], [47]. *Fasciola* spp. are widely endemic across temperate, highland and sub-tropical regions, including parts of Iran, Turkey, China, Nepal, and northern India, where it infects cattle, sheep, and humans [39], [45]. Although *F. gigantica* is generally considered the more prevalent species in many parts of Asia, species-level identification remains challenging when using non-molecular diagnostic approaches. Molecular surveys across East and South Asia have identified overlapping infections with *F. gigantica*, complicating diagnosis and mapping of true *F. hepatica* zones [51], [52]. Human fascioliasis has been documented in China, Vietnam, and Iran, often linked to consumption of aquatic plants harbouring metacercariae [44], [46].

In contrast*, S. japonicum* exhibits a more geographically restricted range, endemic mainly in East and Southeast Asia, especially in China, the Philippines, and small foci in Indonesia [17], [53]. *S. japonicum* is maintained by a wide range of mammalian reservoir hosts (e.g. buffaloes, cattle, pigs, rodents, and dogs) which sustain transmission in rural agricultural settings [35], [36]. Extensive epidemiological studies in China and the Philippines have focused on zoonotic reservoirs, snail intermediate host ecology (classically *Oncomelania* spp.), and integrated control programs [18], [48], [54], [55], [56], [57], [58]*.*

The designation of *S. japonicum* as non-endemic may be misleading and largely reflects the paucity of targeted research in the region. The assumption of absence risks perpetuating a cycle of neglect, whereby perceived non-endemicity discourages investigation and limited data reinforces this perception. This is compounded by substantial gaps in public health surveillance, which rarely include routine screening for schistosomiasis or fascioliasis and often lack molecular diagnostic capacity. In the absence of systematic monitoring, it remains unclear whether current classifications reflect true epidemiology or surveillance deficiencies, underscoring the need for strengthened surveillance and targeted field studies.

Malaysia (Fig. 2) presents a unique ecological and socio-economic setting for *S. japonicum* and *Fasciola* spp., transmission, combining diverse host availability, strong reliance on agriculture, and environmental conditions conducive to parasite life cycles [59], [60], [61], [62]. Despite rapid development, parasitic infections and malnutrition remain prevalent, particularly among indigenous and rural communities [63], [64], [65], [66]. Risk factors vary between Peninsular Malaysia and East Malaysia (Sabah and Sarawak), reflecting differences in agricultural practices, livestock use, water contact, and sanitation infrastructure (Table 2). This highlights the importance of considering zoonotic range, particularly in regional contexts such as Malaysia.

**Fig. 2:**
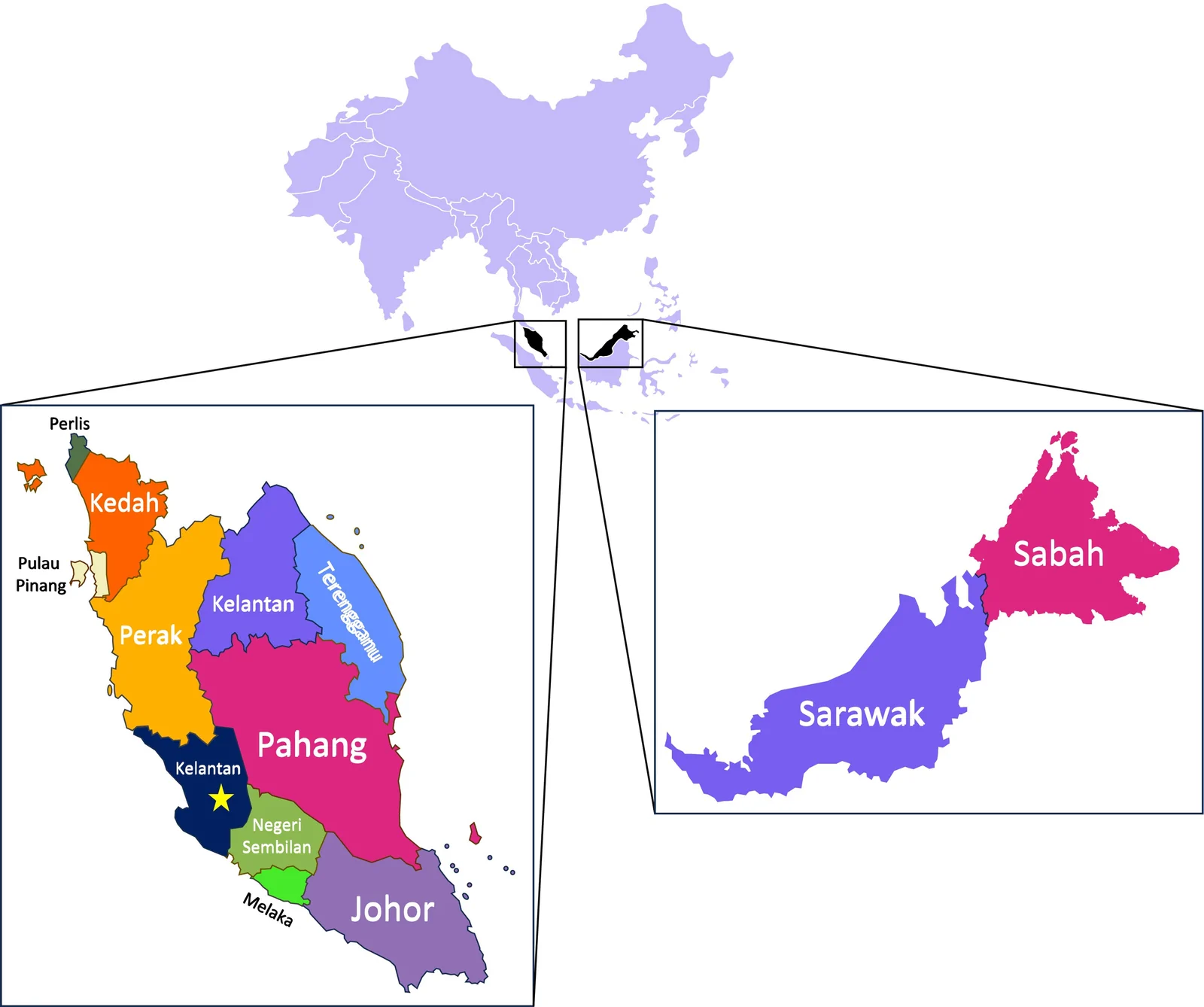
States of Malaysia. Yellow star in left hand box denotes the capital city of Kuala Lumpur. The left box illustrates peninsular Malaysia, and the right, eastern Malaysia.

**Table 2:** Regional breakdown of risk factors for fluke transmission in Malaysia

Factor	Peninsular Malaysia	Sabah & Sarawak
Agricultural Practices	Intensive rice cultivation: high irrigation use creates snail habitats [33], [34].	Lower rice cultivation: oil palm plantations dominate; irrigation canals and stagnant pools from deforestation [64], [67].
Livestock Role	Mechanisation increasing; cattle more common than buffalo [31], [37], [62].	Low mechanisation: water buffalo widely used for draught and transport [62], [68], [69].
Snail Habitat	Irrigation systems and rice paddies favour aquatic snail species [14], [44], [55], [70]*.*	Forest clearance and oil palm irrigation create new snail habitats [67], [71], [72].
Human–Water Contact	Frequent during farming and bathing in irrigation canals [17], [33], [65].	High due to reliance on rivers for bathing, washing, and water collection [17], [65], [66].
Sanitation Infrastructure	Better access in rural areas but still limited in some communities [73].	Poorer infrastructure; shared pit latrines common in indigenous villages [61], [66].
Cultural Practices	Rice farming central to livelihoods; water contact during planting [33], [34].	Communal river use and traditional buffalo farming increase exposure [64], [68].
Risk Summary	Higher risk from agricultural irrigation and human density [63], [70].	Higher ecological and zoonotic exposure risk, driven by livestock reservoirs, shared water use, and limited sanitation infrastructure, rather than documented differences in human prevalence [15], [66].

Evidence from comparable rural areas in China that surveyed 47,139 participants across 47 villages indicates that adult males over 41 years of age, specifically, those engaged in farming, herding, fishing, and boating, are at greatest risk of *S. japonicum* infection due to prolonged occupational water contact [63]. Similar exposure pathways have been reported in the Philippines, where intensive rice cultivation creates extensive interfaces between humans, water buffalo, cattle, and snail-infested irrigation systems [17], [33], [34].

In Malaysia, water buffalo remain culturally and economically important as sources of draught power, meat and milk, however, their frequent contact with water positions them as key reservoir hosts [60], [68]. Shared water sources contaminated by livestock waste further heighten zoonotic transmission risk, as demonstrated in China where bovines contribute up to 75% of *S. japonicum* transmission [31], [60]. Prolonged livestock exposure, limited healthcare access, and poor sanitation in remote areas sustain infection cycles, reinforcing the need for integrated One Health approaches [61].

In Peninsular Malaysia, intensive rice cultivation and widespread irrigation create favourable habitats for aquatic snails, while frequent water contact during planting, weeding, and bathing in canals increases human exposure (Table 2) [17], [33], [34], [62], [63], [65]. Although mechanisation is increasing, cattle species remain widely used, and higher human population density amplifies transmission risk through environmental contamination and repeated exposure [62], [65]. In contrast, Sabah and Sarawak are characterised by lower mechanisation, continued reliance on water buffalo for draught and transport, and extensive use of rivers for bathing, washing, and water collection [17], [62], [64], [65], [66], [67], [68], [69]. Forest clearance and oil palm expansion have created new irrigation channels and stagnant water bodies that serve as snail habitats, while poorer sanitation infrastructure and shared pit latrines in indigenous villages increase environmental contamination [44], [59], [67], [70], [71].

In Sarawak, although rice cultivation is less intensive and often poorly suited to local climatic conditions, it persists because of its social prestige and cultural significance [64]. This suggests that infection patterns comparable to those observed in rural China may occur among Malay and indigenous farmers, although targeted epidemiological studies are needed to confirm this. Close contact with livestock, particularly water buffalo, and reliance on contaminated freshwater sources are recognised risk factors for *Schistosoma* and *Fasciola* infections [65]. High population density further exacerbates transmission by increasing environmental seeding, repeated exposure, and the challenges posed by inadequate sanitation and hygiene practices [64], [66]. In many rural communities, sanitation typically consists of shared single-pit latrines, which have been associated with increased intestinal parasite transmission, as demonstrated among Iban populations in Pakan, Malaysia [66]. Globally*, Fasciola* spp. infections are estimated to cause ∼101–130 disability-adjusted life years (DALYs) per 100,000 people in highly endemic settings; however, Malaysia-specific DALY estimates are unavailable due to limited human surveillance and under-reporting [74], [75]. To date, no robust evidence of endemic *S. japonicum* transmission or reliable prevalence estimates exists in Malaysia, with human cases reported only sporadically. Nevertheless, suitable snail hosts, livestock reservoirs, and shared water use indicate potential ecological risk, while limited surveys, misdiagnosis, subclinical infections, and inconsistent reporting likely lead to underestimation of true prevalence [47], [76]. Chronic fluke infections such as with *S. japonicum* may also predispose individuals to secondary conditions, including colorectal cancer, which often receive greater clinical priority and obscure underlying parasitic disease [77]. Notably, Malaysian public health surveillance data on fluke infections remain sparse, constraining effective control, monitoring, and research.

In Sabah, the presence of both *F. hepatica* and *F. gigantica* has resulted in cattle infection estimates ranging from 6.0% to 48.4%, depending on the year [15]. The current prevalence of *S. japonicum* in Malaysian cattle and water buffalo remains unknown, but data from the Philippines indicate rates of 5.3–22.6% [78], [79]. One study of 480 water buffalo across 14 districts using direct detection methods reported 22.6%, while another study of 57 buffalo in a single district using ELISAs and stool PCRs found a range of 5.2–12.3% [78], [79]. Notably, a qPCR-based study of 105 buffalo from six districts reported prevalence as high as 80.0% [80]. In contrast, a 2020 meta-analysis from neighbouring China reported a prevalence of just 0.2%, reflecting the success of nationwide interdisciplinary control efforts integrating snail control, health education, improved sanitation, and chemotherapy [81].

Together, these patterns highlight the persistence of these neglected tropical diseases in Malaysia and the urgent need for coordinated, cross-sectoral strategies integrating human, animal, and environmental health [1], [4], [7], [10]. Prevalence estimates remain highly uncertain due to variability in diagnostic sensitivity, inconsistent methodologies, and fragmented surveillance. Limited monitoring, misdiagnosis, and underreporting further obscure true infection rates, underscoring the need for harmonised diagnostics and comprehensive nationwide surveys.

In Malaysia, integrated interventions must balance ecological feasibility with cultural and economic realities. Livestock management strategies such as targeted anthelmintic treatment, alongside emerging veterinary vaccines for water buffalo and cattle, are under investigation for reducing reservoir infection [82], [83], [84], [85]. Ecological snail control through agroforestry, biocontrol using ducks or fish, irrigation modification, and earth-filling of stagnant water bodies offers sustainable alternatives to chemical molluscicides [86], [87]. Concurrently, community-driven WASH initiatives, including improved sanitation infrastructure and safe water access, can minimise human exposure during farming and daily activities [88], [89]. These combined measures are cost-effective, culturally adaptable, and achievable within rural agricultural systems, making them the most practical components of a One Health framework for Malaysia.

## 3 Transmission Dynamics: Vectors and Reservoirs

### 3.1 Intermediate Hosts

Both *S. japonicum* and *Fasciola* spp., require intermediate snail hosts: *Oncomelania/Robertsiella/Radix* and *Lymnaeidae*, respectively (Fig. 1). Fewer studies exist in Malaysia, where *S. japonicum* has been sporadically reported but is not under active surveillance, and where *Oncomelania* spp. have not been documented. In these settings, alternative freshwater snail genera such as *Robertsiella* and *Radix* have been identified as intermediate hosts; however, substantially less experimental and field evidence is available regarding their role in transmission compared with *Oncomelania* spp. [13], [14]. Consequently, *Oncomelania* spp. are often retained as a reference intermediate host in comparative discussions, given their well-characterised biology and ecology. Notably, many environmental constraints shaping *Oncomelania–S. japonicum* interactions, including temperature, freshwater habitat availability, seasonal flooding, and climate-driven habitat suitability are shared across amphibious freshwater snail taxa, suggesting that similar ecological pressures, including climate change and land-use intensification, are likely to influence transmission dynamics across different snail host systems [14], [16], [44], [55], [67], [71], [72].

All the host snails aforementioned inhabit slow-moving or stagnant freshwater environments such as ponds, marshes, irrigation canals, and rice paddies, where they facilitate parasite development after miracidia penetrate their tissues [12], [14]. Cercariae (*S. japonicum*) and metacercariae (*Fasciola* spp.) eventually shed and infect definitive host organisms to complete their lifecycles [12]. Due to this reliance on intermediary hosts, the distribution of both *Fasciola* spp., and *S. japonicum* are dependent on the distribution of their obligatory snail intermediary host [41].

Monitoring snail populations remains challenging [90], [91], [92]. Their habitats are often remote or environmentally unstable, such as flooded rice fields or marshlands, and are subject to seasonal fluctuations in water levels, temperature, and vegetation [13]. Snails are small in length, with *Lymnaeidae* species 5-60mm, *O. hupensis* 6-12mm, and *Robertsiella* species ∼<4mm [13], [93], [94], [95]. The snails are highly localised and exhibit rapidly shifting population dynamics complicating consistent surveillance [13]. Within Malaysia, comprehensive nationwide studies are lacking; data focuses on individual states only and has not yet been systematically carried out across all states. This is exemplified in one of the only studies available, estimating an 8.7% cercarial emergence rate, which focuses mainly on snails collected from Perak wetlands [13].

Environmental changes play a critical role in influencing transmission dynamics, especially in tropical regions such as Malaysia. The expansion of agriculture, creation of wetlands, and deforestation provide new habitats for snail vectors [15]. The observed rise in fascioliasis incidence in areas like Ranau may be linked to favourable climatic conditions and the introduction of human-driven agricultural irrigation systems that support the proliferation of snail intermediate hosts [15]. The growing construction of irrigation canals and dams, driven by the expansion of oil palm plantations across Malaysia, is also contributing to this trend [67], [96]. Deforestation alone alters local ecosystems, resulting in the creation of stagnant and slow flowing water bodies that favour the breeding of snail vectors [16], [71], [72]. Compared to undisturbed environments, land clearance alters soil and water chemistry by increasing sunlight exposure and reducing salinity, driving a shift from acidic to neutral or alkaline pH as well as creating new microhabitats [71], [72]. These changes can create optimal new habitats for freshwater snail vectors, increasing their survival and fecundity potential, and thereby elevating the transmission risk of parasitic pathogens [71], [72].

Climate change further exacerbates these challenges by altering the distribution and life cycles of both parasites and their intermediate hosts. Temperature, rainfall, and/or potential evapotranspiration shifts are known to impact parasite development within snails [44]. *Fasciola* spp. egg hatching and miracidia emergence are inhibited below 10.0°C but present a temperature optimum between 20.0–30.0°C, with accelerated cercarial shedding around 27.0°C [97]. Similarly, *S. japonicum* miracidia develops optimally into sporocysts and cercariae at 30.0°C, while development halts below 15.3°C and above 35.0°C [55], [98]. Rising temperatures and altered precipitation may expand snail vector geographical ranges, introducing disease into previously non-endemic areas, including increases in elevational distribution as mountain slopes become warmer.

### 3.2 Reservoir Hosts

Livestock underpin global food systems as demand for animal products rises in developing nations. However, they also act as major reservoirs for zoonotic pathogens, posing significant public health risks [3], [5], [99]. This is especially true for both *S. japonicum* and *Fasciola* spp., which exploit multiple livestock species as hosts (Table 1). Animals often maintain infections until conditions favour human transmission, making sentinel livestock monitoring essential. Risk is shaped by ecological and anthropogenic factors, including habitat loss, biodiversity decline, high species density, pollution, and climate change [100].

Water buffalo represent an overlooked sentinel species in Asia. Two subspecies are recognised: river buffalo (*Bubalus bubalis bubalis*), domesticated in South Asia for milk, and swamp buffalo (*Bubalus bubalis carabanensis*), used in Southeast Asia for rice farming [101]. Of the 200 million buffalo globally, 95% are in Asia [65]. Although often considered disease-resilient (e.g. such as presumed lower mastitis susceptibility due to udder anatomy), these claims are context-dependent and influenced by local environmental factors and management practices [102], [103]. Evidence from China and the Philippines underscores the pivotal role of water buffalo in facilitating fluke transmission to humans [104].

Most epidemiological studies on fascioliasis and schistosomiasis have historically focused on cattle, sheep, and goats. Yet, buffalo pose distinct transmission risks in Malaysia, and it would be remiss to assume a similar symptomology and response due to their known resilience. Buffalo are known to remain asymptomatic for many diseases unlike cattle or sheep, allowing chronic infections to persist undetected [30], [105]. Additionally, unlike other ruminants, their unique wallowing behaviour and reliance on communal water sources increase contact with snail habitats and environmental contamination [60], [68], [69], [104], [106]. Their traditional role as draught animals, rather than for milk or meat, means they are longer-lived and rarely undergo routine veterinary checks if In rural areas, thereby extending their reservoir potential [15], [62], [66], [101]. These traits, combined with cultural and economic importance, position buffalo as potential indicators and major contributors to sustaining transmission cycles.

Sabah hosts approximately 50,000 swamp buffalo [62]. While mechanisation is reducing their role in rice farming, government initiatives now promote buffalo farming for beef production, increasing communal rearing by smallholders [51], [89]. Moreover, maternal genetic diversity of swamp buffalo across Sabah is much lower than other areas in Asia, potentially reducing the adaptability of the livestock in the presence of disease [107]. Despite these factors, their role as sentinel species remains under-investigated.

Buffalo thrive in extensive and semi-intensive systems due to physiological and behavioural adaptations such as wallowing for thermoregulation, dark skin for UV protection, and efficient digestion of low-quality roughage [88], [91], [92]. These traits, however, heighten exposure to *Fasciola* spp*.* and *S. japonicum.* Buffalo’s ability to exploit terrestrial and aquatic environments, combined with unmonitored access to contaminated water and vegetation, makes them key reservoirs for zoonotic pathogens. For instance, in northern India, feral buffalo interact with domestic and wild *Bubalus arnee* populations, facilitating cross-species transmission [108]. In China, buffaloes have been implicated in the resurgence of *S. japonicum* transmission in areas where schistosomiasis had previously been controlled [32].

Of particular concern is the effect of *Fasciola* spp. infection in cattle, which suppresses immune responses, reducing the sensitivity of interferon-gamma (IFN-γ) assays used for detecting bovine tuberculosis (bTB), increasing false negatives [109]. This is concerning in regions like Selangor, where bTB persists despite ongoing culling efforts [[110], [111]].

In Pakistan, limited freshwater access forces buffalo to bathe in wastewater and sewage, greatly increasing pathogen transmission and human infection risk [[112], [113]]. In Malaysia, water-use patterns remain poorly studied, hindering accurate risk assessments for rural communities. Targeted disease monitoring programs, such as those in Italy, have successfully reduced bTB prevalence in buffalo, underscoring the value of surveillance in mitigating zoonotic threats [114]. Despite legal requirements for reporting *Fasciola* spp. and *S. japonicum* infections, practical enforcement challenges, particularly beyond large commercial farms, may allow infections to go unreported, reducing the effectiveness of disease tracking and intervention [47]. Testing for both parasites in livestock, particularly buffalo, is infrequent and not standardised. Compounding these challenges, Malaysia’s tropical climate supports year-round snail habitats, while irrigation and livestock-water contact perpetuate transmission. These ecological dynamics underscore the urgent need for integrated environmental management alongside medical interventions.

Given the interconnectedness of human, animal, and environmental factors in parasite transmission, a One Health approach is essential. Integrated strategies combining environmental management, improved sanitation, and livestock health measures offer sustainable solutions to reducing infection rates. This framework strengthens rural resilience and ensures interventions are scientifically sound, socially equitable, and economically viable.

## 4 The One Health Perspective

### 4.1 Diagnostic Issues

Accurate detection of *Fasciola* spp. and *S. japonicum* is essential for identifying reservoirs, guiding interventions, and sustaining transmission control. Despite the availability of multiple diagnostic approaches, significant challenges remain in achieving sensitive, specific, and operationally feasible surveillance across both human and animal hosts (Table 3). In Malaysia, human schistosomiasis caused by *S. japonicum* dates to 1975 among the Orang Asli population; however, few epidemiological studies have since been conducted, leaving critical knowledge gaps [115]. The diversity of diagnostic tools and their uneven application across sectors continue to constrain effective control.

**Table 3:** Comparative analysis of diagnostic methods for zoonotic flukes: benefits, limitations, and applicability.

Method	Principle	Detection Limit	Benefits	Limitations	Cost	Applicability	Species Specificity
Kato-Katz / FEC/ FlukeFinder® Kit	Microscopic detection of eggs in faeces or rapid sedimentation and isolation of eggs	Detects only patent infections; low sensitivity for <50 eggs/g faeces [101], [102], [103]	Quick (≈10 min/sample);Low cost, simple, widely used; non-invasive; suitable for field use [[116], [117]].	Reduced sensitivity in low-intensity or early infections; egg output variability; species misidentification; false negatives post-treatment [101], [102], [103], [104]. Diagnostic thresholds and egg output differ substantially between *Schistosoma* spp. and *Fasciola* spp.	Very Low (few pence per slide) [116]. FlukeFinder**®** £2-5 per kit	Kato-Katz used for human diagnosis. FEC & FlukeFinder® are primarily animal use.	Poor – relies on egg morphology; hybrids complicate identification [[82], [117]].
Necropsy (post-mortem)	Direct visual inspection of liver and bile ducts	Detects heavy infections only; cannot detect immature stages [117].	Highly accurate for severe infections; useful in abattoirs; low cost [117].	Invasive; only applicable post-slaughter; cannot detect early infections [117].	Low [117].	Animals only	Moderate – adult morphology helps, but hybrids may confuse identification [117].
Radiological (CT, MRI, Ultrasound)	Imaging of hepatic lesions and fibrosis patterns	Detects chronic pathology; cannot confirm active infection without serology [[33], [73], [118], [119], [120], [121], [122]].	Non-invasive; rapid; useful for chronic schistosomiasis (e.g., periportal fibrosis) [[33], [121]].	High cost; requires trained personnel; limited rural access; false positives (lesions mimic neoplasms); impractical for livestock [[33], [73], [118], [119], [120], [121], [122]].	Very High (£200–£800 per scan) [[118], [119], [123]].	Humans (rarely Animals)	None – imaging alone cannot confirm species [121].
Serological (ELISA, IHA)	Detects host antibodies or circulating antigens	Detects infections from ∼2 weeks post-infection; cannot distinguish past vs active [[54], [124], [125], [126], [127], [128], [129]]	High sensitivity; useful for screening; detects subclinical infections [117].	Cannot confirm active infection; species-specific validation needed; blood sampling logistics; qualitative only [[54], [124], [125], [126], [127], [128], [129]]	Moderate (£5–£20 per test) [[126], [127], [129]].	Humans & Animals	High – requires species-specific antigen panels; cross-reactivity possible [[126], [127], [128], [129]].
Molecular (PCR, qPCR, LAMP)	Detects parasite DNA in faeces or blood	Detects very low parasite loads; prepatent detection possible [[51], [52], [80], [117]]	High specificity; detects hybrids and novel species; useful for epidemiology [[80], [117]].	Expensive; requires specialised equipment and personnel; longer processing time; limited routine veterinary use [[51], [52], [80], [117]]	High (£30–£100 per sample) [51], [52], [80].	Humans & Animals	Excellent – species and even strain-level identification possible [51], [52].

Diagnostic methods can be broadly grouped into direct parasitological techniques, radiological imaging, serological/immunological assays, and molecular approaches (Fig. 3). Each differs in principle, sensitivity, cost, and applicability, resulting in marked variability in performance (Table 3). This variability is particularly problematic where surveillance is primarily human-focused, potentially overlooking animal reservoirs that sustain zoonotic transmission.

**Fig. 3:**
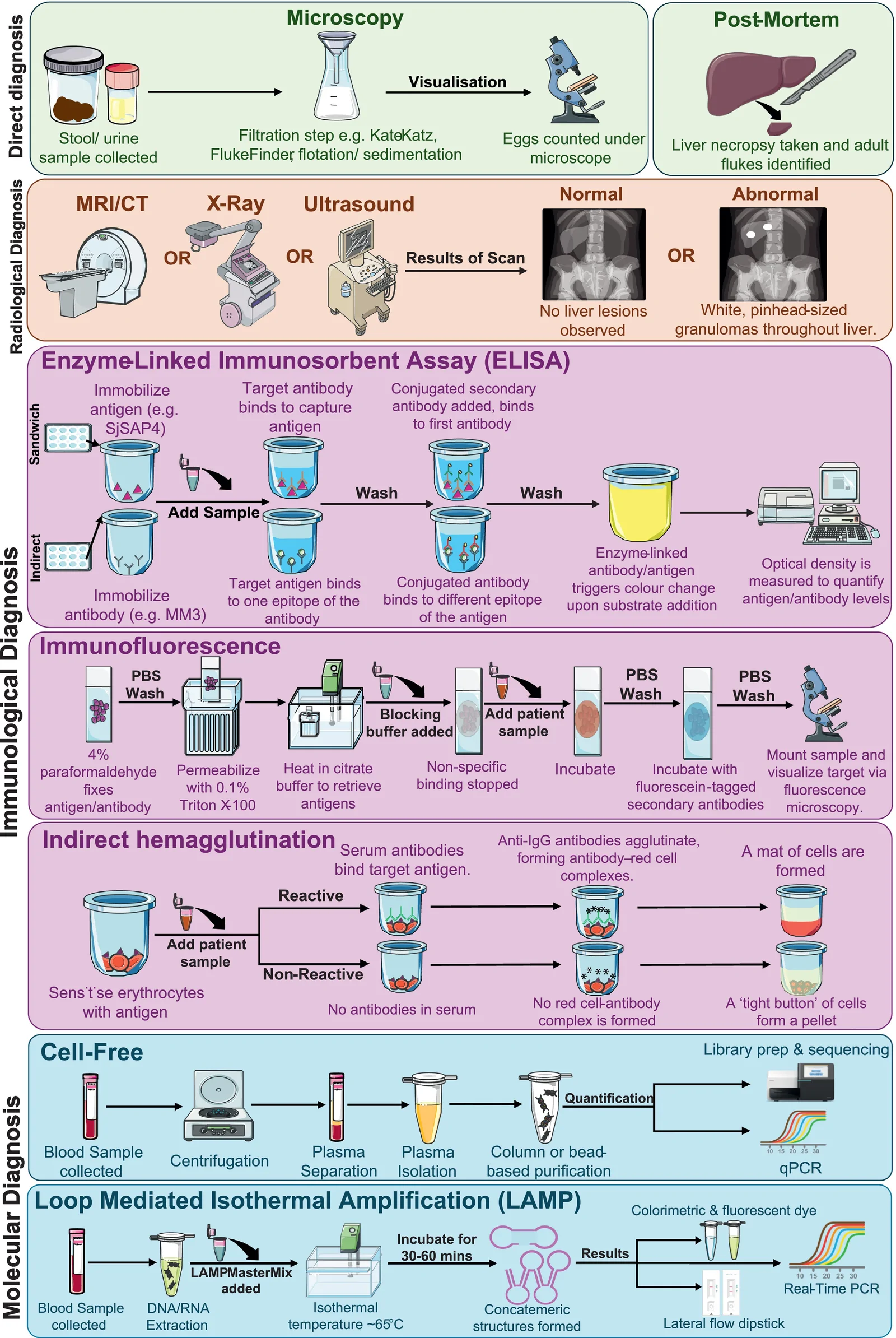
Methods of fluke detection in final hosts. Green segments indicate direct diagnosis methods, orange radiological, purple immunological and blue molecular. Abbreviations: magnetic resonance imaging (MRI), computed tomography (CT). Figure created using elements from Servier Medical Art [49].

Traditional direct methods, including Kato-Katz (human diagnosis), faecal egg counts (FEC) (mainly *Fasciola* spp*.,* but can be used for *S, japonicum)*, and post-mortem inspection, remain widely used in animal diagnostics because they are inexpensive, simple, and field-adaptable [116]. Innovations such as the Fluke-Finder® kit improve livestock diagnostic efficiency, enabling rapid egg isolation (≈10 minutes per sample) with minimal training [116]. Although primarily used for *Fasciola* spp., it can also detect *S. japonicum* eggs; however, it is not validated for human samples and shows reduced sensitivity in low-burden infections [[116], [130]]. In abattoir settings, necropsy remains the primary method for diagnosing fascioliasis and identifying liver condemnation due to its low cost and minimal infrastructure needs [48]. However, all direct techniques depend on mature flukes in hepatic tissues that are able to shed eggs, making them insensitive to early-stage, low-intensity, or prepatent infections [[19], [115]]. Consequently, prevalence is frequently underestimated.

Egg-based diagnostics are further compromised by daily variation in egg shedding, host immune responses, prior treatment, and difficulties in species discrimination, particularly in the presence of hybrids [64], [101]. While FEC correlates reasonably with fluke burden in *Fasciola* spp., it does not reliably reflect adult *Schistosoma* worm loads [102], [104]. Age-related immunity in livestock can suppress egg output, requiring sampling over 10% of a herd across age groups to improve accuracy [101].

Post-treatment egg retention in the gall bladder may also produce false negatives [117]. Prevalence rates are often underestimated, demonstrated in a study in China involving over 47,000 humans where Kato-Katz identified a 1.9% *S. japonicum* prevalence compared to 11.9% by ELISA [63]. Underestimation of infection impedes timely intervention and weakens control programmes [47].

Radiological imaging, including ultrasound, CT, and MRI, offers rapid, non-invasive assessment of chronic pathology. In schistosomiasis, ultrasonography is valuable for detecting periportal fibrosis, splenomegaly, and portal hypertension [[121], [123]]. However, imaging cannot confirm active infection or identify species, and parasitic lesions may mimic neoplasms, risking misdiagnosis and inappropriate management [[118], [119], [120]]. High equipment costs and the need for specialised personnel limit accessibility, particularly in rural Malaysia where patients may need to travel considerable distances to tertiary centres [61], [73]. In veterinary contexts, radiological diagnosis is rarely feasible due to infrastructure constraints, logistical challenges, and the impracticality of imaging large animals under field conditions [[122], [131]].

Serological and immunological assays provide higher sensitivity than direct methods and are useful for screening and detecting subclinical infections. Nonetheless, antibody-based tests cannot reliably distinguish between past and active infections, potentially inflating prevalence estimates [47]. Test performance varies with host biology, antigen clearance, protein binding, and serum composition, while faecal antigen detection may be influenced by gut physiology and microbiome differences [124]. Circulating antigen assays, although sensitive, may show reduced specificity in low-transmission settings [125]. Importantly, each assay requires validation for the specific host species, as interspecies differences in antibody responses and antigen dynamics can compromise sensitivity and specificity [[54], [117]]. Distinct immunoglobulin profiles in humans (dominance of IgG1 and IgG4) and livestock (broader isotype response of IgG2 and IgE) further necessitate careful optimisation and interpretation [[126], [127], [128], [129]]. Practical challenges in blood collection and processing also limit routine use in veterinary surveillance, and the qualitative nature of many assays restricts assessment of infection intensity.

Molecular diagnostics offer high sensitivity and specificity, enabling detection of low parasite burdens, hybrids, and novel species such as *Schistosoma malayensis* [117]. These tools enhance epidemiological resolution and understanding of host–parasite interactions. However, their high cost, requirement for specialised equipment and trained personnel, and longer processing times limit routine application, particularly in livestock and resource-limited settings [117]. As with other non-direct methods, sample collection, storage, and transport can affect accuracy, and methodological variability between laboratories complicates comparability.

Across all approaches, a major constraint is the fragmented nature of diagnostic practices, which are typically developed and applied separately in human and veterinary sectors. This siloed approach reduces efficiency, hinders data integration, and limits understanding of zoonotic transmission. A One Health framework would enable co-development and cross-validation of diagnostic tools, harmonised sampling strategies, and shared use of specialised infrastructure, improving surveillance coherence and cost-effectiveness.

Given the limitations of high-cost molecular and imaging techniques in rural Malaysia, routine surveillance should prioritise low-cost, field-adaptable methods. For livestock, sedimentation-based FEC, Mini-FLOTAC or FlukeFinder kits offer practical, rapid screening options with minimal infrastructure [[116], [117]]. In humans, Kato-Katz remains the most practical for community screening despite lower sensitivity, and can be complemented by ELISA in sentinel populations to improve accuracy [17], [63]. These approaches align with One Health principles by enabling scalable, integrated monitoring across human and animal hosts in resource-limited settings.

### 4.2 Integrated Control Strategies

Both *Fasciola* spp. and *S. japonicum* are treatable infections and their control relies on integrated strategies combining chemotherapy (e.g. triclabendazole for fascioliasis, praziquantel for schistosomiasis), environmental management, snail control, and improved sanitation. In Malaysia, effective and sustainable control requires a comprehensive One Health approach that links environmental, veterinary, agricultural, and community-based interventions (Fig. 4). Given that both parasites share transmission pathways, intermediate snail hosts, and ecological risk factors, there is substantial scope for synergistic, cross-sectoral collaboration. Integrating human and animal health surveillance and control programmes offers an opportunity to overcome long-standing disciplinary silos and enhance efficiency.

**Fig. 4:**
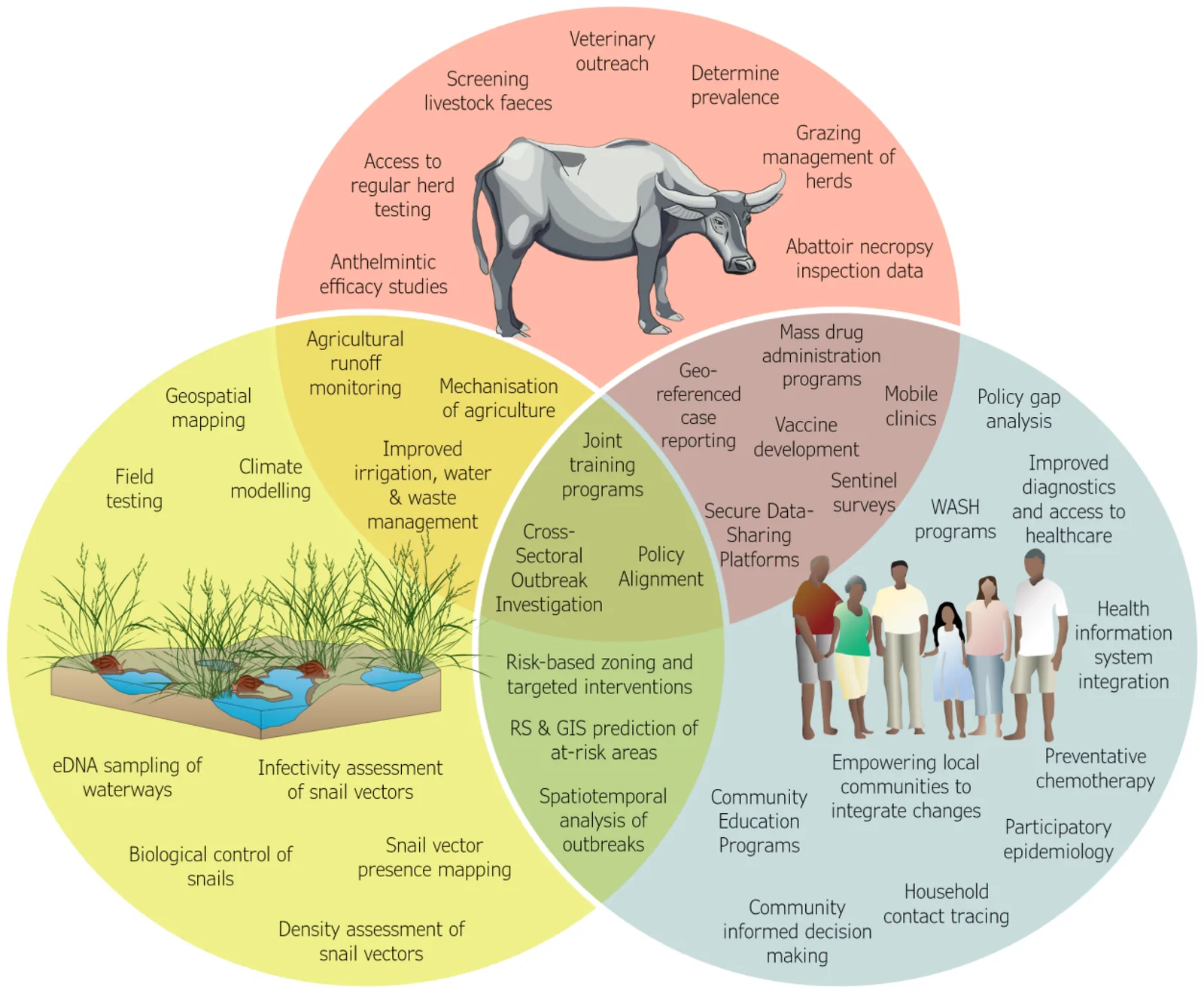
A One Health approach to controlling *Schistosoma japonicum* and *Fasciola* spp*.* in Malaysia. A Venn diagram to show the cross-sectionalism of monitoring and surveillance, epidemiology, and integrated control strategies. The top (red) is livestock based, left (green) is environmental & snail, right (blue) is human health. Abbreviations are as follows: RS = remote sensing, GIS = Geographic Information Systems, WASH = Water, Sanitation and Hygiene. Figure created by the authors using elements from the Integration and Application Network [50].

Modern surveillance tools, including remote sensing (RS) and geographic information systems (GIS), are increasingly valuable for identifying high-risk areas and guiding targeted interventions. Environmental markers such as temperature, surface water, and vegetation indices can be used to predict snail habitat suitability and disease hotspots [88]. GIS-based risk mapping has been successfully applied in schistosomiasis-endemic regions in Egypt, improving community engagement and informing intervention strategies [132]. Incorporating these technologies into Malaysian public health surveillance could strengthen targeted delivery of control measures in rural endemic settings.

Bovine hosts, particularly Water Buffalo, remain central to the transmission of both parasites. While full mechanisation of agriculture has reduced transmission in parts of China, Malaysia’s lower mechanisation levels necessitate alternative strategies such as livestock vaccination [37], [88]. Recent trials of *S. japonicum* vaccines demonstrated significant reductions in worm burden and transmission, and experimental approaches show potential for mitigating fascioliasis in cattle [82], [84], [85]. Ecological interventions, including agroforestry, upland–paddy rotation, and habitat modification, have been effective in reducing snail populations in nearby economically comparable countries and are more sustainable than chemical molluscicides, which pose risks to aquatic ecosystems [[88], [133]]. In China, long-term use of fast-growing tree plantations in endemic areas eliminated snail populations through changes in soil moisture, pH, and vegetation cover [133]. Biocontrol approaches, such as introducing snail-predatory ducks and fish, also offer environmentally friendly alternatives [[48], [86], [134]]. Community-driven action is essential, integrating health education, participatory mapping, and local engagement. Coordinated environmental, veterinary, agricultural, and social interventions within a One Health framework underpin sustainable fascioliasis and schistosomiasis control in Malaysia.

## 5 Concluding Remarks and Future Perspectives

Implementing a One Health approach to control *S. japonicum* and *Fasciola* spp. in Malaysia offers clear benefits but faces major challenges. Collaboration across public health, veterinary, environmental, and agricultural sectors is critical but often hindered by institutional silos, resource constraints, and competing priorities [135]. One of the most significant barriers currently, is the lack of comprehensive epidemiological data. Livestock infections are documented, with prevalence reaching up to 95% in some cattle farms, yet human fascioliasis remains severely underreported, and schistosomiasis surveillance is virtually absent [[47], [136]]. Although *S. japonicum* is not currently endemic, the presence of competent snail hosts, changing land-use patterns and increased livestock movement raise concerns about future transmission [13].

To address underreporting, practical improvements in clinical screening are essential. In resource-limited healthcare settings, stool-based sedimentation techniques remain the most cost-effective initial tool, but their low sensitivity for early infections warrants complementary methods. We recommend ELISA-based serology for suspected cases and community screening, and coproantigen detection where feasible for active infections. Molecular assays (PCR, LAMP) should be reserved for reference laboratories and outbreak investigations. Training healthcare workers to recognise atypical hepatobiliary symptoms and including fascioliasis in differential diagnoses for chronic liver disease will further improve detection. For livestock, nationwide surveillance should prioritise sedimentation-based faecal egg counts and Mini-FLOTAC kits, which are rapid, inexpensive, and field-adaptable. These should form the backbone of routine monitoring, supplemented by serological assays in sentinel herds for improved sensitivity. The strengthening of surveillance systems, expanding diagnostic capacity, and fostering intersectoral collaboration will be critical to reducing the burden of these neglected parasites and safeguarding both human and animal health.

Effective control requires interventions feasible in rural settings. Ecological snail control through agroforestry, irrigation modification, and earth-filling of stagnant water bodies offers sustainable alternatives to chemical molluscicides, while biocontrol using ducks or fish can complement these measures [[86], [133]]. Strategic anthelmintic treatment of cattle and water buffalo, along with community-driven WASH programs can reduce human exposure [[85], [116]].

Public awareness of the zoonotic aspect remains low among rural communities, healthcare providers, and travellers, with 75% unaware of the snail intermediate host [[137], [138]]. While attitudes toward treatment, prevention, and control are generally positive, most communities lack access to healthcare [137]. Culturally relevant education campaigns are essential but require sustained collaboration and funding. Malaysia must strengthen surveillance, expand diagnostics, improve intersectoral communication, and implement community-based education. Urgent research is needed on transmission ecology, host-parasite interactions, and resistance patterns. A robust One Health framework is critical to reducing *S. japonicum* and *Fasciola* spp. burdens and improving health outcomes for humans and animals.

## CRediT authorship contribution statement

**Jessica Wyn Brocklebank:** Writing – review & editing, Writing – original draft, Visualization, Resources, Investigation, Funding acquisition, Formal analysis, Data curation, Conceptualization. **Luke Davies:** Writing – original draft, Resources, Conceptualization. **Benoît Goossens:** Writing – review & editing, Funding acquisition, Conceptualization. **Laura Braun:** Writing – review & editing, Funding acquisition. **Pablo Orozco-terWengel:** Writing – review & editing, Supervision, Funding acquisition, Conceptualization.

## Declaration of competing interest

The authors declare that they have no known competing financial interests or personal relationships that could have appeared to influence the work reported in this paper.

## Data Availability

No data was used for the research described in the article.
